# Modeling breathing rhythms

**DOI:** 10.7554/eLife.46033

**Published:** 2019-03-25

**Authors:** Jan-Marino Ramirez, Nathan A Baertsch

**Affiliations:** 1 Center for Integrative Brain ResearchSeattle Children's Research InstituteSeattleUnited States; 2 Department of Neurological SurgeryUniversity of WashingtonSeattleUnited States; 3 Department of PediatricsUniversity of WashingtonSeattleUnited States

**Keywords:** breathing rhythm, non-selective cation current, computer modeling, persistent sodium current, rhythmogenic circuits, rhythmogenic kernel, None

## Abstract

Computational models are helping researchers to understand how certain properties of neurons contribute to respiratory rhythms.

**Related research article** Phillips RS, John TT, Koizumi H, Molkov YI, Smith JC. 2019. Biophysical mechanisms in the mammalian respiratory oscillator re-examined with a new data-driven computational model. *eLife* **8**:e41555. doi: 10.7554/eLife.41555

Orchestral music is beautiful, rich and complex, yet analyzing the contribution of any one instrument can be difficult. In many ways, neuroscientists face similar challenges when they try to understand how the networks in the brain work. These circuits are made of many types of neurons, each of which has different properties. Moreover, the properties of a neuron can change because of its interactions with other cells in the network.

Researchers have started to unravel this complexity by building computational models of both neurons and networks of neurons, and by focusing on pairs of interacting properties. This is similar to how one might study how the left and right hand of a violinist work together to produce melody and rhythm, and then use this knowledge to better understand other stringed instruments and their roles in the orchestra.

Within the ‘rhythmogenic’ networks of the brain, different types of neurons work together to create the body rhythms that are essential for life. For example, a complex network generates breathing rhythms, and it is often used to understand rhythmogenic circuits in general. The balance between excitatory and inhibitory connections between neurons is critical to shape network activity (Ramirez and Baertsch, 2018). Equally important, but less well understood, are the interactions between intrinsic properties that are built into individual neurons.

In particular, two intrinsic properties are thought to play a role in controlling breathing rhythms and network activity in general. The first is a persistent sodium current (I_NaP_), which is slowly activated and inactivated by changes in the voltage across the neuronal membrane. The second does not depend on voltage: rather, the calcium-activated non-selective cation current (I_CAN_) is triggered when the level of calcium ions inside the cell increases. The source of these calcium ions is unknown, but there is experimental evidence that they could be provided by mechanisms at the synapses between neurons (Del Negro et al., 2010; Del Negro et al., 2011). Both I_NaP_ and I_CAN_ allow some neurons to generate rhythms on their own, without being stimulated by their neighbors. Yet, in the actual network, these properties also enhance the signals transmitted by excitatory synapses (Ramirez et al., 2004). Because I_NaP_ and I_CAN_ interact with synaptic properties, as well as with each other, it is difficult to isolate their relative contributions.

Pharmacological and genetic manipulations have shed light on how I_NaP_ and I_CAN_ work in the respiratory network (see, for example, Koizumi et al., 2018; Picardo et al., 2019), but there is still no consensus on how they contribute to rhythmogenesis (Feldman and Del Negro, 2006; Ramirez et al., 2004). Now, in eLife, Jeffrey Smith of the National Institute Neurological Disorders Stroke (NINDS) and colleagues – including Ryan Phillips as first author – report results from a computational modeling approach that re-examines how I_NaP_ and I_CAN_ control breathing (Phillips et al., 2019).

The researchers, who are based at NINDS, the University of New Hampshire and Georgia State University, established a simplified model of the breathing network, which only takes into account excitatory synaptic interactions. Then, they tuned the contributions of three variables, I_NaP_, I_CAN_, and the source of intracellular calcium ions, and explored how this affected the frequency and amplitude of breathing. First, they examined how changes in the source of calcium ions influenced the contribution of I_CAN_ to the network. If calcium came from within neurons, I_CAN_ controlled the frequency, but not the strength, of breathing. On the other hand, if calcium depended on synaptic activity, I_CAN_ acted as a synaptic amplifier to control the strength of the rhythm, but it had little effect on its frequency. This scenario best matched experimental data and prior conclusions (Del Negro et al., 2011; Koizumi et al., 2018), prompting Phillips et al. to conclude that I_CAN_ is activated by calcium ions that are primarily of synaptic origin.

The group then went on to demonstrate that I_CAN_ does not establish the rhythm in their computational model, but that it increases the strength of breathing by recruiting more neurons to participate in the network. Indeed, when I_CAN_ was eliminated from the model network, a weak breathing rhythm remained, which was generated by a small number of neurons with high levels of I_NaP_ activity (Figure 1). Eliminating this ‘rhythmogenic kernel’ from the model stopped the rhythm altogether, in agreement with some (Peña et al., 2004), but not all (Pace et al., 2007) experimental data.

**Figure 1.**
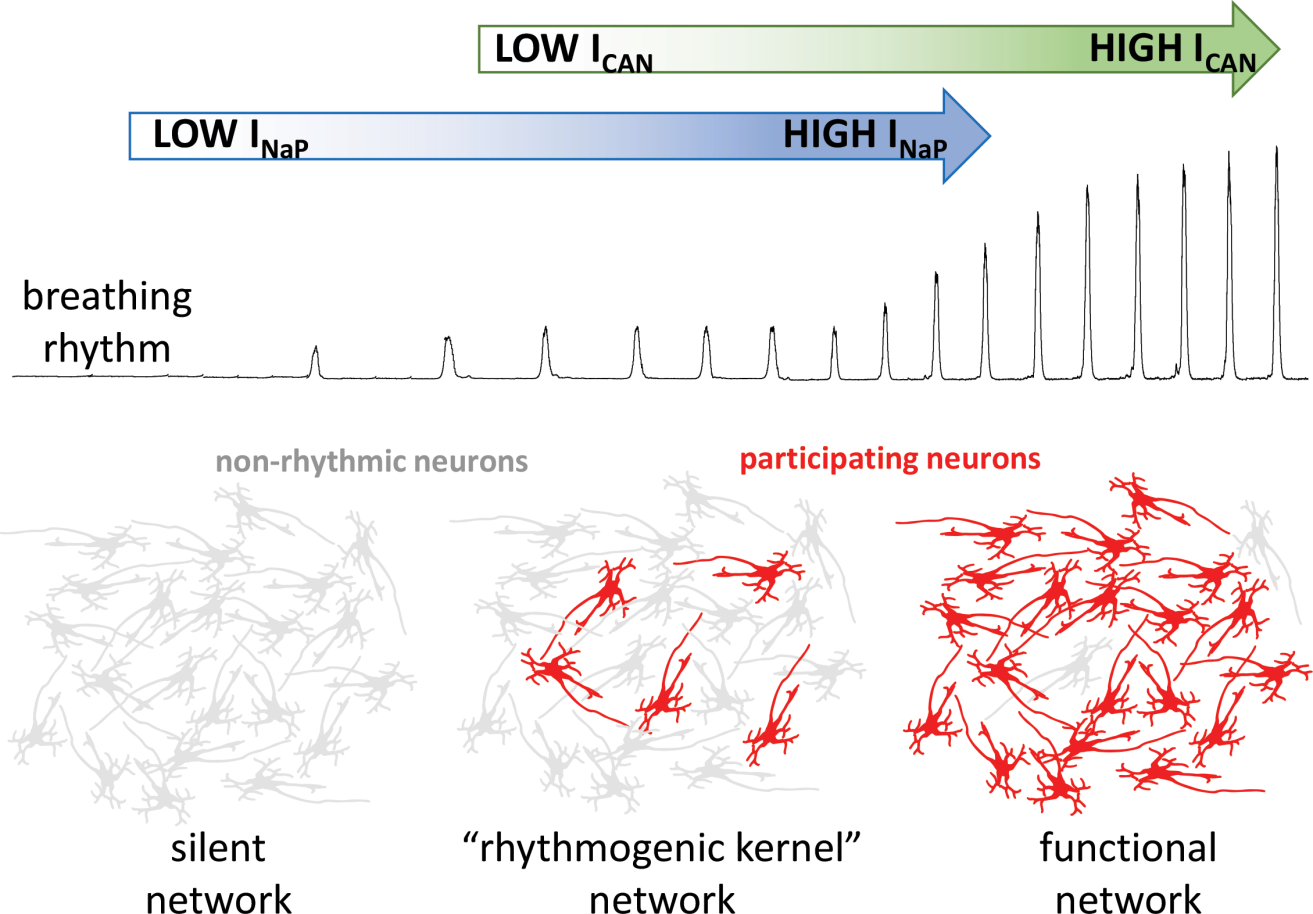
How the intrinsic properties of neurons contribute to the breathing rhythm. Phillips et al. established a model of the brain network that controls breathing, and used it to deduce how two built-in properties of neurons, the I_NaP_ sodium current (blue) and the I_CAN_ current (green), control the breathing rhythm. Without I_NaP_, the network is silent, with the neurons exhibiting non-rhythmic activity (grey). However, a small number of neurons with high levels of I_NaP_ activity can produce a weak rhythm, even in the absence of I_CAN_. In turn, the model suggests that I_CAN_ is activated by calcium ions coming from synapses; the current would then amplify excitatory interactions between neurons. This amplification leads to additional neurons participating in the rhythm (red), producing a robust functional network.

Unlike the model built by Phillips et al., the actual network that controls breathing is not exclusively excitatory, but is subjected to important inhibitory and neuromodulatory control. Further, alternative mechanisms of rhythmogenesis that do not depend on I_NaP_ have also been proposed (Del Negro et al., 2010; Rubin et al., 2009). Could different rhythmogenic mechanisms therefore contribute to breathing, depending on the demands of the network? In particular, could the contributions of I_NaP_ and I_CAN_ change based on the neuromodulatory state of the network? Addressing such questions will require further back-and-forth between experiments and increasingly complex models.

Overall, the results of Phillips et al. allow us to understand how intrinsic neuronal properties independently control the strength and frequency of the breathing rhythm. Their model is also a useful framework in which to explore how changes in the way I_NaP_ and I_CAN_ interact can dynamically impact rhythmogenic properties. For example, it could shed light on the way how the network reconfigures when the body lacks oxygen. Ultimately, describing the duet between I_NaP_ and I_CAN_ in the respiratory network may help dissect how rhythmic activity is controlled in other regions of the brain (Penn et al., 2016; Riquelme et al., 2018).
